# Prevalence of persistent parasitic infections in foreign-born, HIV-infected persons in the north of Spain

**DOI:** 10.7448/IAS.15.6.18105

**Published:** 2012-11-11

**Authors:** A Rodriguez-Guardado, C Suarez, M Fernandez, M Rodriguez, O Martinez, S Rojo, F Perez

**Affiliations:** 1 Hospital Universitario Central de Asturias, Infectious Diseases Unit, Oviedo, Spain; 2 Hospital Universitario Central de Asturias, Microbiology Unit, Oviedo, Spain

## Abstract

**Background:**

Foreign-born, HIV-infected persons are at risk for sub-clinical parasitic infections acquired in their countries of origin. This study presents the results of this screening program.

**Methods:**

A prospective, descriptive study was designed to include all the immigrant patients diagnosed of HIV infection attending in Hospital Central de Asturias, Spain, 2006–2011. We included demographic variables, CD4+cells count and viral load at time of diagnosis. Screening comprised blood count, biochemistry, basic urinalysis, hepatitis B virus (HBV), HCV, strongyloidiasis and schistosomiasis serologic analysis, stool parasites, blood test for filarias, PCR for malaria and Chagas disease serologic analysis and PCR in persons from Latin America. Qualitative variables were compared using the χ^2^ test, the Fisher exact test, when necessary. For quantitative variables, the Student t test for nonpaired variables or the Mann-Whitney U test were used. Significance was designated at p<0.05.

**Results:**

57 patients were analyzed. 70% are sub-Saharan immigrant and the rest Latin American. The most frequent countries of origin were Equatorial Guinea (43%), Nigeria (10%), Senegal (9%), Colombia (9%). Average time in Spain: 1,061 days (3–9,876). Average Cd4+cells were 209 cells/mm^3^. The average viral load were 47,000 RNA viral copies. Intestinal parasites were diagnosed in 27 patients: *T. trichuria* (22%), strongyloidiasis (11%), amebiasis (7%), and schistosomiasis (5%), *G. intestinalis* (4%). All infections by *T. trichuria* were diagnosed in Equatorial Guinea patients. Other parasites diseases were: filariasis by *M. perstans* (9%); malaria (9%, all from Equatorial Guinea), Chagas disease (4%). Eight patients had chronic hepatitis B virus and 2 patients had HCV hepatitis. 19% of patients had latent syphilis, significantly more frequent in sub-Saharan patients (9 vs 2; p=0.04). In 12 patients the screening did not show any disease.

**Conclusions:**

Given the high prevalence of certain parasite infections and the potential lack of suggestive symptoms and signs, selected screening for strongyloidiasis and schistosomiasis or use of empiric antiparasitic therapy may be appropriate among foreign-born, HIV-infected patients. Identifying and treating helminth infections could prevent long-term complications.

